# Role of umbilical cord mesenchymal stromal cells in skin rejuvenation

**DOI:** 10.1038/s41536-024-00363-1

**Published:** 2024-05-10

**Authors:** Le Chang, Wei-Wen Fan, He-Ling Yuan, Xin Liu, Qiang Wang, Guang-Ping Ruan, Xing-Hua Pan, Xiang-Qing Zhu

**Affiliations:** The Basic Medical Laboratory of the 920th Hospital of Joint Logistics Support Force of PLA, The Transfer Medicine Key Laboratory of Cell Therapy Technology of Yunan Province, The Integrated Engineering Research Center of Cell Biological Medicine of State and Regions, Kunming, 650032 Yunnan Province China

**Keywords:** Stem-cell research, Ageing

## Abstract

Aging is the main cause of many degenerative diseases. The skin is the largest and the most intuitive organ that reflects the aging of the body. Under the interaction of endogenous and exogenous factors, there are cumulative changes in the structure, function, and appearance of the skin, which are characterized by decreased synthesis of collagen and elastin, increased wrinkles, relaxation, pigmentation, and other aging characteristics. skin aging is inevitable, but it can be delayed. The successful isolation of mesenchymal stromal cells (MSC) in 1991 has greatly promoted the progress of cell therapy in human diseases. The International Society for Cellular Therapy (ISCT) points out that the MSC is a kind of pluripotent progenitor cells that have self-renewal ability (limited) in vitro and the potential for mesenchymal cell differentiation. This review mainly introduces the role of perinatal umbilical cord-derived MSC(UC-MSC) in the field of skin rejuvenation. An in-depth and systematic understanding of the mechanism of UC-MSCs against skin aging is of great significance for the early realization of the clinical transformation of UC-MSCs. This paper summarized the characteristics of skin aging and summarized the mechanism of UC-MSCs in skin rejuvenation reported in recent years. In order to provide a reference for further research of UC-MSCs to delay skin aging.

## Introduction

The skin is the largest organ of the human body and has a surface area of 1.5–2 m^2^, covering the surface of the human body. It is in direct contact with the external environment and protects us from environmental factors^1^. The skin consists of three parts: the epidermis, dermis, and subcutaneous tissue, which jointly protect internal organs and perform different biological functions. The epidermis is located in the outermost layer of the body and plays a major defensive role^2^. The dermis is mainly responsible for the synthesis, deposition, and remodeling of the dermal extracellular matrix (ECM), which supports the structural integrity of the skin^3,4^. Dermal fibroblasts are the main cells in the dermis and synthesize and secrete collagen, elastin and proteoglycan to give strength and elasticity to the skin^5^. Subcutaneous tissue is located in the deepest layer of the skin and is rich in fat cells and blood vessels; it can support, warm, and provide nutrition for the dermis^6^. Skin appearance is the main factor used to evaluate age and health status. With the emergence of aging complications and the improvement in quality of life, people are highly motivated to maintain a youthful appearance. Therefore, how to prevent and delay skin aging is important for the general public, thereby stimulating the in-depth study of antiaging by researchers.

Under aging and the decline in the structure and function of skin tissue stimulated by external factors, many functional cells in skin tissue undergo senescence and apoptosis, while new cells lack the ability to self-renew. To resolve the insufficient self-renewal ability of cells in skin tissue, some researchers have proposed that skin cells can be replenished by activating stem cells in skin tissue. However, long-term activation and mobilization will lead to the depletion of stem cells in the body and the complete loss of the ability of cells in the skin to self-renew^7^. It has been reported that MSCs transplantation can improve skin conditions to some extent^8^. Therefore, exogenous supplementation of MSCs may be an effective method. The term “MSCs” originated from the isolation of the bone marrow in 1988 Marrow Stromal Stem Cells^9^, and named Mesenchymal Stem cells by A.I. Caplan in 1991^10^, ISCT changed to Mesenchymal Stromal Cells in 2006^11^, A.I. Caplan himself applied to ISCT in 2017 to change Mesenchymal Stem Cells to Medicinal Signaling Cells^12^, ISCT stated in 2019 that it was not in favor of dropping the term “mesenchymal” and recommended that the acronym “MSC” continue to be used, but with a note on the functional definition^13^. The naming of MSCs is still controversial, but with further research, increasing evidence suggests that the therapeutic role of MSCs is largely attributed to their paracrine function.

In this paper, we focus on the study of UC-MSCs in skin aging. On the one hand, umbilical cords are medical waste, and as a result, using them avoids the limitation of source and ethical issues^14–18^. On the other hand, the efficacy of MSCs decreases with the increase of their number of divisions, because cell division shortens telomeres and leads to cell senescence^19,20^. An earlier 2012 follow-up study of patients with the acute graft-versa-host disease (GVHD) treated with MSCs showed a significant increase in one-year survival (75% vs 21%) in MSCs receiving early passage (from generation 1–2) compared to MSC patients receiving late passage (from generation 3–4)^21^. In addition, the regenerative potential of MSCs also decreases with the age of donors^22^. Therefore, the UC-MSCs are isolated from neonatal tissues and seem to be “younger” than other sources of MSCs^23^. Their high activity, increased pluripotency, low immunogenicity, and suitable paracrine effects have been indicated^24,25^. Previous studies have shown that UC-MSCs can be induced to differentiate into various types of functional cells in vitro, such as keratinocytes and dermal fibroblasts, which provides a variety of potential strategies for the treatment of skin diseases and the development of medical beauty products^26^. It also made many researchers once believe that the efficacy of MSCs is mainly played by their differentiation into specific functional cells, so that the efficacy of MSCs is infinitely amplified, resulting in over-marketing of “Stemcells” on the market. However, subsequent studies have seen little evidence that MSCs can differentiate into specific functional cells in vivo, so it is believed that the paracrine role of MSCs is the main way to exert their therapeutic effect. This may also be the reason why A.I. Caplan applied to ISCT in 2017 to change Mesenchymal Stem Cells to Medicinal Signaling Cells.

### Characteristics of skin aging

Human skin is a dynamic and complex organ that is composed of different cell types and functional regions. Like other organs, the skin ages and is characterized by structural destruction and gradual loss of function. Aging caused by genetic, metabolic, and other internal factors is called intrinsic aging, while aging caused by environmental factors such as ultraviolet rays, nutrition, air pollution, cigarettes, temperature, and pressure is called extrinsic aging^27^.

For naturally aging skin, histological changes mainly occur in the basal layer and dermis. The basal layer of the skin is located in the deepest layer of the epidermis and participates in the repair and regeneration of the skin. Studies have shown that the proliferation of basal cells of the skin, such as keratinocytes and melanocytes, decreases with age, resulting in a thinning of the skin epidermis^28,29^. Moreover, the fibrous ECM components such as elastin, fibrin, and collagen in the dermis degenerate, the skin is dehydrated, elasticity decreases, and wrinkles appear^30,31^. With age, the repair ability of skin cells decreases, resulting in intrinsic skin aging.

Extrinsic aging is far more serious than endogenous aging; ultraviolet radiation (UVR) has the greatest effect, accounting for 80% of facial skin aging^32^. In contrast to intrinsic aging, UVR thickens the epidermis and promotes the activation of epidermal melanocytes in exposed skin, resulting in pigmentation^33^. UVR on the skin leads to senescence and apoptosis of skin cells by directly damaging the deoxyribonucleic Acid (DNA), Ribonucleic Acid (RNA), and protein of skin cells^34^. Moreover, skin cells produce free radicals and reactive oxygen species (ROS) when subjected to UVR, which causes inflammation and promotes mitochondrial membrane potential (MMP) synthesis, indirectly leading to oxidative damage and ECM degradation of skin cells^35,36^. Photoaging also accelerates skin aging by superimposing intrinsic aging in chronological order.

The ultimate goal of researchers’ efforts investigating skin aging is to find ways to slow down the rate of aging and improve quality of life by regulating the mechanisms of skin aging. At present, it has been reported that plant extracts^37^, antioxidants^38^, growth factors, and cytokines^39^, as well as MSCs^40^, can alleviate skin aging^41^. Since cell therapy was first proposed by Swiss doctors in 1931, the field has made a breakthrough in the research of human diseases. Skin tissue is composed of a large number of mature functional cells, progenitor cells, and a small number of stem cells. Although adult tissue stem cells are rare, they play a major role in human health. The number of adult stem cells gradually decreases after birth, so supplementation with exogenous MSCs may be an effective way to promote tissue repair and regeneration. Umbilical cord mesenchymal stromal cells (UC-MSCs) have become a more promising therapeutic method because of their powerful paracrine function and the ability to secret various cytokines, growth factors, and exogenes to promote tissue regeneration and inhibit inflammatory response. However, MSCs therapy is still in the research stage, and a large amount of experimental data is needed to accelerate its clinical transformation. As of December 2023, over 2000 MSCs clinical trials have been registered at https://clinicaltrials.gov/, including over 400 UC-MSCs clinical trials. Including UC-MSCs for Diabetic Nephropathy, Ulcerative Colitis, Oral Chronic Graft-versus-host Disease, Diabetic Foot, Skin Grafts in Donor Site Wounds, Skin Rejuvenation, Skin Ulcers, and other diseases. This large amount of data reflects the broad interest of the scientific community in the potential therapeutic applications of MSCs. However, among the many clinical trials at different stages, we have collated nine clinical trials of UC-MSCs for skin-related diseases that have been completed and have reported results (Table 1). By combing through these trials, we can gain a clearer understanding of the application of UC-MSCs in clinical practice, as well as the challenges and future directions. Clinical trials are designed to evaluate the efficacy and safety of MSCs in the treatment of various diseases, but clinical trials currently face many difficulties, including developing standardized treatment protocols, monitoring cell survival and function in vivo, and the safety and long-term efficacy of cell therapy. These problems not only increase the complexity of clinical trials, but also limit their wide application in practice. In order to solve the challenges faced by clinical trials, pre-clinical basic research is crucial to provide a reliable theoretical and experimental basis for clinical trials. In the basic research, the establishment of an ideal experimental model is the premise of further research, here we mainly introduce the skin aging research model. Aging research on animal models can simulate the complex environment of human skin aging in combination with in vitro and in vivo aging factors and relatively accurately reflect the characteristics of skin aging, but the accuracy of these results still needs to be verified at the cellular and molecular levels. Cells are the basic unit of the human body; they can be isolated and expanded in vitro under suitable conditions and can reflect the process and law of human aging at the cellular level, so they are widely used as an experimental model in vitro. In order to facilitate the work of subsequent researchers, we have listed in detail the modeling conditions of the currently widely used research models in Table 2, aiming to provide clearer and convenient guidance for future basic research. However, it is worth noting that none of these studies calculated the percentage of actual engrafted cells relative to the total implanted cells, as the actual number of engrafted cells is crucial for assessing therapeutic efficacy. Therefore, future research may need to pay more attention to and carefully consider the calculation of the actual number of engrafted cells to comprehensively understand the effectiveness and mechanisms of MSC therapy.

**Table 1:** Reported clinical trials using UC-MSCs for the treatment of skin-related disorders

Year	Disease	Interventions	Patient count	Efficacy
2022^85^	Psoriasis	Intravenous infusion of UMSCs (1–3 × 10^6^ cells/kg)	17	A total of 47.1% (8/17) of the psoriasis patients had at least 40% improvement in the PASI score, and 17.6% (3/17) had no sign of disease or minimal disease based on the PGA score.
2020^86^	Cesarean section skin scars	Intravenous infusion of UMSCs low-dose (3 × 10^6^ cells) high-dose (6 × 10^6^ cells)	90	UC-MSCs did not demonstrate the effects of improvement of cesarean section skin scars
2020^61^	Atopic dermatitis (AD)	USC-CM containing cream	28	USC-CM may have therapeutic effect for AD as cosmetics and drug materials
2019^87^	Facial rejuvenation	RCE cream	40	Aging facial skin is significantly improved
2021^88^	Recessive dystrophic epidermolysis bullosa (RDEB)	Intravenous infusion of hUCB-MSCs (1–3 × 10^6^ cells/kg)	6	hUCB-MSC administration induced M2 macrophage polarization and reduced mast cell infiltration in RDEB skin. Serum levels of substance P were decreased after therapy.
2020^89^	Skin of patients treated with AFL	USC-CM containing serum and cream	23	The application of human cord blood cells containing serum and cream resulted in accelerated wound healing and reduced post-treatment erythema.
2017^90^	Atopic dermatitis (AD)	hUCB-MSCs subcutaneous injection low dose (2.5 × 10^7^) high dose (5.0 × 10^7^)	34	A single treatment of hUCB-MSCs resulted in dose-dependent improvements in AD manifestation
2016^91^	Diabetic Foot	Intravenous injection of UC-MSCs around foot ulcers	53	After 3 months of treatment, new blood vessels increased significantly, and the ulcer healed completely or gradually.
2019^92^	Chronic skin ulcers	Covering wounds with acellular amniotic membrane seeded with WJSCs	5	The wound healing time and wound size significantly decreased

**Table 2:** Establishment method and evaluation metrics of the skin aging model

Model type	Concrete model	Intervention	Metrics
Animal	Lanyu pigs^93^, guinea pigs^94^, nude mice^95^, hairless dogs^96^, Rats^97^, C57BL/6 N mice^98^	Age-associated senescent, nicotine, UVB, D-Galactose	(1) Epidermal and dermal thickness (2) Ki67, P16, P53, P21, MDA, SOD, ROS, HYP, GSH, AGEs, collagen I, collagen III, elastin and HA levels (3) SA-β-Gal staining
Cell	HaCaT cells^99^, HFF^100^, HSF^101^, HDFs^102^	Replicative senescence, IL-17, tBHP, H_2_O_2,_ UVB, D-Galactose	(1) Cell viability and migratory properties (2) IL-6, TNF-α, MMP-1, MMP-2, MMP-9, TIMP-1, TIMP-2, LaminB1, pRB, Ki67, p53, p21, p-p53, γ-H2AX, ROS levels (3) SA-β-Gal staining

### The role and mechanism of UC-MSCs in skin aging

**Fig. 1:**
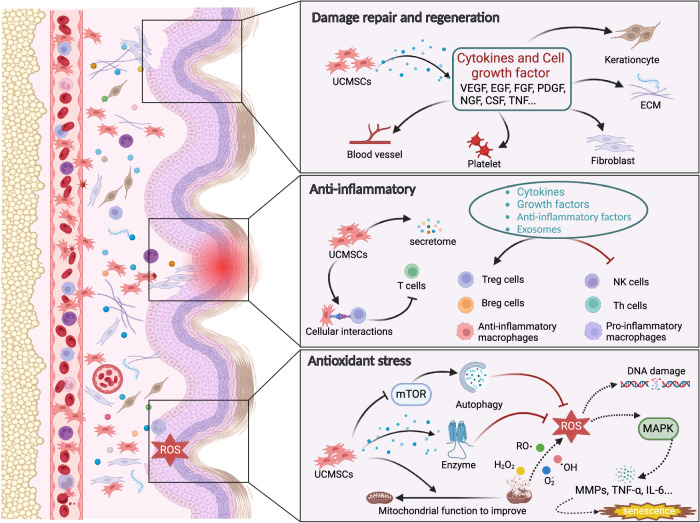
The role and mechanism of UC-MSCs in skin senescence. The skin shows structural and functional degradation under the action of internal and external factors, and UC-MSCs rejuvenate it by promoting injury repair and regeneration through anti-inflammatory, antioxidative, and anti-glycosylation mechanisms.

UC-MSCs are a kind of mesenchymal stromal cell derived from neonatal umbilical cord tissue with abundant material sources, easy amplification, strong plasticity, low immunogenicity, high migration and homing activity, exocrine secretion, and the secretion of a variety of cytokines^25^. Compared with other MSCs currently used in basic and clinical research, such as adipose mesenchymal stromal cells (ADMSCs), bone marrow mesenchymal stromal cells (BMSCs), dental pulp stromal cells (DPSCs), embryonic stromal cells (ASCs), and neural stem cells (NSCs); UC-MSCs are derived from a wider range of sources; have no ethical or safety challenges; are easier to obtain, expand and store; and can fully meet clinical needs^42^. UC-MSCs can be induced to differentiate into many types of functional cells in vitro, which is of great significance for the clinical treatment of corresponding diseases. UC-MSCs have been used in the study of cardiovascular disease^43^, inflammatory bowel disease (IBD)^44^, chronic obstructive pneumonia (COPD)^45^, premature ovarian failure (POF)^46^, skin aging^23^, and other diseases, and their effectiveness has been proven. This paper mainly summarizes the research progress of UC-MSCs in skin aging. The mechanism of UC-MSCs in the treatment of skin aging can be summarized as promoting injury repair and skin regeneration through anti-inflammatory, antioxidative, and anti-glycosylation mechanisms, as shown in Fig. 1.

### Damage repair

Skin tissue integrity, function, and regeneration decrease with age. An increasing number of studies have reported that UC-MSCs can promote the repair of damaged skin through the secretion of cytokines. The homing property of UC-MSCs is the key to their direct participation in the repair of skin injury. Many animal experiments have confirmed that when there is injury in the body, transplanted UC-MSCs can migrate to the injured site, differentiate, and replace injured cells using the chemotaxis of the injured tissue microenvironment^47–49^. However, with the deepening of the research, the view that MSCs differentiate and replace injured cells is no longer supported. After the importation of MSCs into the body, the amount of MSCs in the body is very small (<1%), suggesting that the repair of injuries may primarily involve the paracrine functions of MSCs (Table 3).

**Table 3:** The mechanism of UC-MSCs in skin rejuvenation in the last 5 years

Model	Delivery strategy	Findings	Mechanism	Reference
Nude mice	Subcutaneous injection of hUCMSCs (0.25–25 × 10^4^ cells)	(1) After hUCMSCs treatment from low to high doses, the skin wrinkles of nude mice gradually faded. (2) hUCMSCs restored the SOD, MDA, Col-1, and VEGF levels. (3) hUCMSCs increase the dermal thickness and production of collagen fibers.	hUCMSCs removed wrinkles and smoothened skin texture by exerting antioxidative and regenerative effects on skin ECM.	^23^
SD rats	Multi-point subcutaneous injection of hUCMSCs (1.6 × 10^5^ cells)	(1) hUCMSCs promoted the wound healing of irradiation-induced skin ulcers. (2) hUCMSCs promoted keratin generation and cell proliferation of irradiated area.	hUCMSCs improved ulcer healing via *PI3K/Akt* pathway.	^103^
BALB/C mice	Intravenous injection of UC-MSC (1 × 10^6^ cells)	(1) UC-MSCs treatment ameliorated bleomycin‑induced skin fibrosis. (2) UC-MSCs treatment reduced local inflammation (3) UC-MSCs treatment displayed an anti‑fibrotic effect by regulating Th17 cell activation in the impaired skin.	UC-MSCs treatment exerted an anti‑fibrotic role by alleviating local inflammation and Th17 cell activation in the impaired skin	^104^
SD rats	Femoral vein injection of hUCMSC (5 × 10^6^ cells)	(1) hUCMSCs accelerated wound healing in DFU rats by promoting epithelialization, granulation tissue formation collagen deposition, and angiogenesis. (2) hUCMSCs had the ability to modulate the synthesis of several cytokines and chemokines, which are involved in the inflammatory process to restrain the inflammatory reaction.	Transplanted hUCMSCs via intravenous injection improved wound healing in the DFU rat model through paracrine action and trans-differentiation.	^105^
HDFs	Cultured in CMM with hUCMSCs	(1) CMM significantly restored the wound area ratios. (2) The number of SA-β-gal stained senescent HDFs was decreased. (3) Senescence-related genes of HDFs were restored.	The anti-senescent paracrine mechanism of hucMSCs was the inhibition of autophagy in senescent HDFs.	^23^
HaCaT cells	Cultured in hUCMSCs-exo(600 µg)	(1) hUCMSCs-exo reduced ROS production. (2) hUCMSCs-exo protected skin keratinocytes from oxidative stress. (3) hUCMSCs-exo delivered 14-3-3ζ protein, which promoted *SIRT1* expression and activated autophagy to alleviate HaCaT cell damage under oxidative stress conditions.	hUCMSCs-exo upregulated *SIRT1* levels in HaCaT cells by trafficking 14-3-3ζ protein to relieve UV radiation and H_2_O_2_-induced oxidative stress damage.	^106^
Dermal fibroblast	Cultured in UC-MSC-EVs (10 µg/ml)	(1) EVs from TGFβ-stimulated UC-MSCs induced fibroblast proliferation and migration. (2) Total collagen and elastin were increased by the stimulation of EVs from TGFβ-stimulated UC-MSCs.	EVs from UC-MSCs protected skin from aging by promoting ECM protein production.	^107^
HDFs	Cultured in medium containing GDF (0.1 µg/ml)	(1) UC-MSCs secreted cytokines that stimulated HDFs growth. (2) UC-MSCs expressed and secreted high levels of GDF-11, which promoted HDFs migration and ECM production in vitro. (3) UC-MSCs-CM in cosmetic products increased dermal density and decreased skin wrinkles in humans.	UC-MSCs-CM has various useful growth factors, including GDF-11, that can stimulate skin rejuvenation by increasing growth and ECM production of HDFs.	^108^

To study the role and fate of transfused MSCs, Yin’s research team explored the fate of type 2 diabetes (T2DM) mice intravenously injected with UC-MSCs compared with that in control mice. This study showed that UC-MSCs first reached the lungs and then migrated through the circulatory system to the spleen and liver. Compared with the control mice, the T2DM mice injected with UC-MSCs showed that the UC-MSCs homed to the islets. UC-MSC infusion not only effectively restored blood glucose homeostasis and reduced insulin resistance in mice but also improved hyperlipidemia and liver function in T2DM mice, suggesting that UC-MSC migration is closely related to tissue injury and can participate in tissue repair^50^. Zhang et al.^51^ applied UC-MSCs and UC-MSC-CM locally to the skin wounds of diabetic mice to study their therapeutic effects on wound healing. The results showed that UC-MSCs and UC-MSC-CM significantly increased the overall wound healing rate, improved angiogenesis, and increased the percentage of M2 macrophages in the wound area. Further observation of the local microenvironment of the wound tissue showed that the secreted levels of the anti-inflammatory factors IL-10 and VEGF increased, while the secreted levels of the proinflammatory factors TNF-α and IL-6 were inhibited. It is suggested that UC-MSCs can play a role in injury repair by improving angiogenesis and regulating the local tissue microenvironment.

### Promotion of skin regeneration

“Repair” and “regeneration” are often mistaken for the same concept. In fact, “repair” mainly refers to the recovery of tissue structure and function. In the context of skin, repair indicates that it may have scars and may not have hair follicles. “Regeneration” essentially refers to achieving a completely normal state through the proliferation of cells in skin tissue^52^. UC-MSCs can secrete and synthesize a variety of cytokines that promote cell growth and differentiation to regulate the local microenvironment, including FGF, EGF, VEGF, NGF, PDGF, CSF, and TNF^53^. These cytokines carry signaling information that can regenerate blood vessels, improve blood circulation, and promote tissue regeneration.

After skin injury model rats were treated with UCBMSC-exo and UCBMSCs, the skin appendages, blood vessels, and nerves were regenerated, the wound closure rate was significantly accelerated, and scarring was reduced^54^. Li et al.^23^ used an aging nude mouse model and HDF model to prove that UC-MSCs can increase the thickness of aging skin and the production of matrix collagen fibers, increase the proliferation and migration of human dermal fibroblasts (HDFs), and promote skin regeneration. In addition, an interesting study showed that UC-MSCs can also be used as carriers for gene transfer and drug delivery to enhance the expression of the target gene and can interact with cytokines to change the secretion level to enhance regeneration. The Wnt protein is the key mediator of skin development. Researchers obtained conditioned medium (Wnt-CM) containing Wnt7a from the supernatant of UC-MSCs overexpressing Wnt7a and injected it into the wounds of mice. It was found that the supernatant promoted wound healing, induced hair follicle regeneration, and enhanced the expression of the ECM and the migration of fibroblasts^55^.

### Anti-inflammation

Inflammation is a pathophysiological reaction after tissue injury and a protective defense response of tissues and organs to harmful stimuli. A certain degree of inflammation is beneficial, but excessive inflammation can lead to local tissue cell necrosis and dysfunction, and persistent chronic inflammation can hinder the growth or regeneration of functional cells in tissue^56,57^. Moreover, the human body is always exposed to various stimuli, and long-term inflammatory stimulation eventually leads to the degeneration of the structure and function of tissues and organs. Therefore, the reduction in inflammatory reactions may be beneficial to the regeneration of tissues and organs. Experiments showed that the gradual accumulation of senescent cells in the body increased the release of proinflammatory factors such as IL-6, IL-8, and TNF-α and further promoted the occurrence of senescence^58^. Photoaging is the main form of skin aging. Long-term exposure to UVR accelerates the aging of skin under the action of inflammatory cells and proinflammatory cytokines^59^.

UC-MSCs exert their anti-inflammatory effect mainly by secreting cytokines, growth factors, anti-inflammatory factors, and exocrine factors to reduce the inflammatory response and enhance tissue repair. They can also directly interact with the surface molecules of immune cells and regulate the downstream pathways of immune cells, thus affecting cell proliferation, effector production, and cell survival^60^. Several Korean researchers have used antibody arrays to evaluate the concentrations of growth factors and cytokines in UC-MSC-CM. The results showed that UC-MSC-CM contained high concentrations of anti-inflammatory-related growth factors and cytokines, including EGF, TIMP-1, IGFBP-7, thrombin reactive protein-1, fibrinogen, and fibronectin^61^. The authors further tested the anti-inflammatory activity of UC-MSCs-CM on HaCaT cells stimulated with TNF-α and INF-γ. The results showed that UC-MSC-CM had an inhibitory effect on the inflammatory cytokines TARC, TNF-β, IL-1β, and IL-6 and suggested that UC-MSC-CM had an anti-inflammatory effect. Li et al.^62^ confirmed that UCMSC treatment can reduce the expression levels of the proinflammatory cytokines TNF-α, IL-1β, and IL-6 in an LPS-induced rat model and concluded that UCMSC treatment can reduce systemic inflammation associated with LPS.

We know that continuous inflammation stimulates tissue fibroplasia, leading to tissue and organ fibrosis. Liu et al.^63^ used a rat model of renal interstitial fibrosis to evaluate the effect of UCMSC-CM on tubulointerstitial inflammation and fibrosis. The results showed that UCMSC-CM reduced the deposition of ECM, the infiltration of inflammatory cells, and the release of inflammatory factors in renal fibrosis by inhibiting the activation of the *TLR4/NFκB* signaling pathway. Chen’s team^64^ injected UC-MSCs subcutaneously into psoriatic arthritis model mice and found that UC-MSCs inhibited skin inflammation and significantly ameliorated the pathological features of mice.

### Antioxidant properties

Oxidation is a process in which substances are decomposed to release energy and take place in the body regularly. When the body is in a normal physiological state, the oxidation capacity and antioxidant capacity are in dynamic balance. Once the production of free radicals (such as ROS) exceeds the body’s antioxidant capacity, the redox state is out of balance, and oxidative stress is induced^65,66^. Oxidative stress is accompanied by the processes of cell injury, inflammation, and metabolic disorders, which are involved in the pathology of many diseases and are considered to be the cause of aging. It has been reported that excessive ROS can directly oxidize DNA, proteins, and lipids, resulting in DNA damage, mitochondrial damage, protein damage, cell senescence, and even death^67–69^. According to the theory of free radical aging, ROS are mainly produced as a result of cell metabolism dysfunction and UVR; are generated by the mitochondrial electron transport chain, peroxisomes, and endoplasmic reticulum; and play a major role in skin aging^70^. ROS can activate the *MAPK* signaling pathway through a series of intermediates to promote the production of MMPs. MMPs can degrade collagen and elastin, resulting in increased and deepened skin wrinkles and a lack of elasticity^71^. ROS and the activated *MAPK* signaling pathway can also activate *NFκB*, mediate the expression of inflammatory cytokines, further promote the production of ROS, and accelerate skin aging^72,73^.

There are few reports on the antioxidant effect of UC-MSCs on skin aging. Some scholars believe that UC-MSCs can directly alleviate mitochondrial dysfunction, thus blocking the production of more free radicals from dysfunctional mitochondria that accelerate aging, but the specific mechanism is not clear^74^. However, an increasing number of researchers have observed the antioxidant stress effect of UC-MSCs in aging animal models. Recently, it was reported that after UCMSC treatment of D-galactose-induced skin aging model nude mice, the levels of superoxide dismutase (SOD) in skin tissue increased significantly, while the levels of malondialdehyde decreased significantly, essentially returning to normal levels^23^. It is suggested that UCMSC treatment can enhance the ability of cells to scavenge free radicals, improve the antioxidant stress function of skin, and play a positive role in reducing cell senescence caused by oxidative stress. However, while the antioxidant effects of UCMSC treatment have been observed, the underlying mechanism is still not completely clear. Some scholars believe that UC-MSCs play an antioxidant stress role by directly scavenging free radicals, secreting bioactive enzymes, and regulating the function of mitochondria, but there is insufficient evidence^75^.

### Anti-glycosylation

Advanced glycation end products (AGEs) are the products of nonenzymatic glycosylation and oxidation of proteins and lipids, which accumulate in inflammatory environments and during aging. The accumulated AGEs easily interact with collagen fibers in the dermis to produce glycosylated collagen in the body. The structural changes of glycosylated collagen increase skin fragility and decrease skin strength so that its biological function is reduced^76,77^. A new study showed that UC-MSCs can protect fibroblasts from AGE cytotoxicity by secreting cytokines and activating the *PI3K/AKT/PTEN* pathway^78^.

Senile diabetes is a very common chronic disease related to aging. The difficulty of healing skin wounds in patients with diabetes is a problem that urgently needs to be solved^78^. An in-depth study of the pathogenesis of diabetic dermatopathy found the root cause of diabetic wound formation and healing difficulty to be the accumulation of AGEs in the dermis. However, to date, only a few effective methods can inhibit and remove AGEs in wounds, and the emergence of MSCs therapy brings great hope to these diabetic patients^79–82^. Many researchers have studied the promoting effect of mesenchymal stromal cells from different sources on diabetic wound healing. The results show that BMSCs and UC-MSCs can effectively promote diabetic skin wound healing^83,84^.

## Methods

### Data sources

This review conducted extensive searches across PubMed, ClinicalTrials.gov using the search terms “umbilical mesenchymal stromal cells,” “skin aging,” “regeneration,” “rejuvenation,” “mechanism,” “review,” “clinical trial,” and “retrospective study” to retrieve relevant reviews and studies published within the past 20 years. A total of 108 eligible literature pieces were screened.

### Inclusion criteria

The selection criteria are as follows: 1. Literature discussing the nomenclature, biological characteristics, and mechanisms of action of MSCs in depth; 2. Articles involving experiments and clinical studies on UC-MSCs in promoting skin regeneration and repair; 3. Literature covering the construction of skin aging models and MSC treatment strategies; 4. High-quality literature provides rigorous explanations of facts and viewpoints.

### Exclusion criteria

The exclusion criteria are lack of relevance, repetitive studies, and outdated articles.
